# Pathogenic *Neisseria* Bind the Complement Protein CFHR5 *via* Outer Membrane Porins

**DOI:** 10.1128/iai.00377-22

**Published:** 2022-10-04

**Authors:** Wearn-Xin Yee, Christoph M. Tang, Hayley Lavender

**Affiliations:** a Sir William Dunn School of Pathology, University of Oxford, Oxford, United Kingdom; Stanford University

**Keywords:** *Neisseria gonorrhoeae*, *Neisseria meningitidis*, complement, CFH, CFHR

## Abstract

Neisseria meningitidis and Neisseria gonorrhoeae are important human pathogens that have evolved to bind the major negative regulator of the complement system, complement factor H (CFH). However, little is known about the interaction of pathogens with CFH-related proteins (CFHRs) which are structurally similar to CFH but lack the main complement regulatory domains found in CFH. Insights into the role of CFHRs have been hampered by a lack of specific reagents. We generated a panel of CFHR-specific monoclonal antibodies and demonstrated that CFHR5 was bound by both pathogenic *Neisseria* spp. We showed that CFHR5 bound to PorB expressed by both pathogens in the presence of sialylated lipopolysaccharide and enhanced complement activation on the surface of N. gonorrhoeae. Our study furthered our understanding of the interactions of CFHRs with bacterial pathogens and revealed that CFHR5 bound the meningococcus and gonococcus *via* similar mechanisms.

## INTRODUCTION

The complement system is a key component of the innate immune system and is critical for the recognition and elimination of invading microorganisms. Complement can be activated by three main pathways, the classical, lectin, and alternative pathway (AP) (reviewed in reference (1)), with all the pathways generating a C3 convertase which cleaves C3 into C3a and C3b. C3b then binds to the activating surface leading to opsonization with or without lysis through the terminal complement pathway (2). As C3b binds nonspecifically to both host and pathogen molecules, complement activation must be tightly regulated to prevent host cell damage (3). Complement factor H (CFH) is the major negative regulator of the AP and inhibits complement activity by preventing the formation of the AP C3 convertase or accelerating its decay, and by acting as a cofactor for C3b cleavage mediated by factor I. CFH consists of 20 Complement control protein modules (CCPs) which each consist of approximately 60 amino acids (4, 5). The four N-terminal CCP domains of CFH (CFH_[1-4]_) possess complement regulatory activity, while other domains, such as CFH_(7)_ and CFH_(19-20)_, enable interaction with ligands such as glycosaminoglycans, sialic acid found on host cells and the C3 activation fragment, C3b (6, 7).

**FIG 1:**
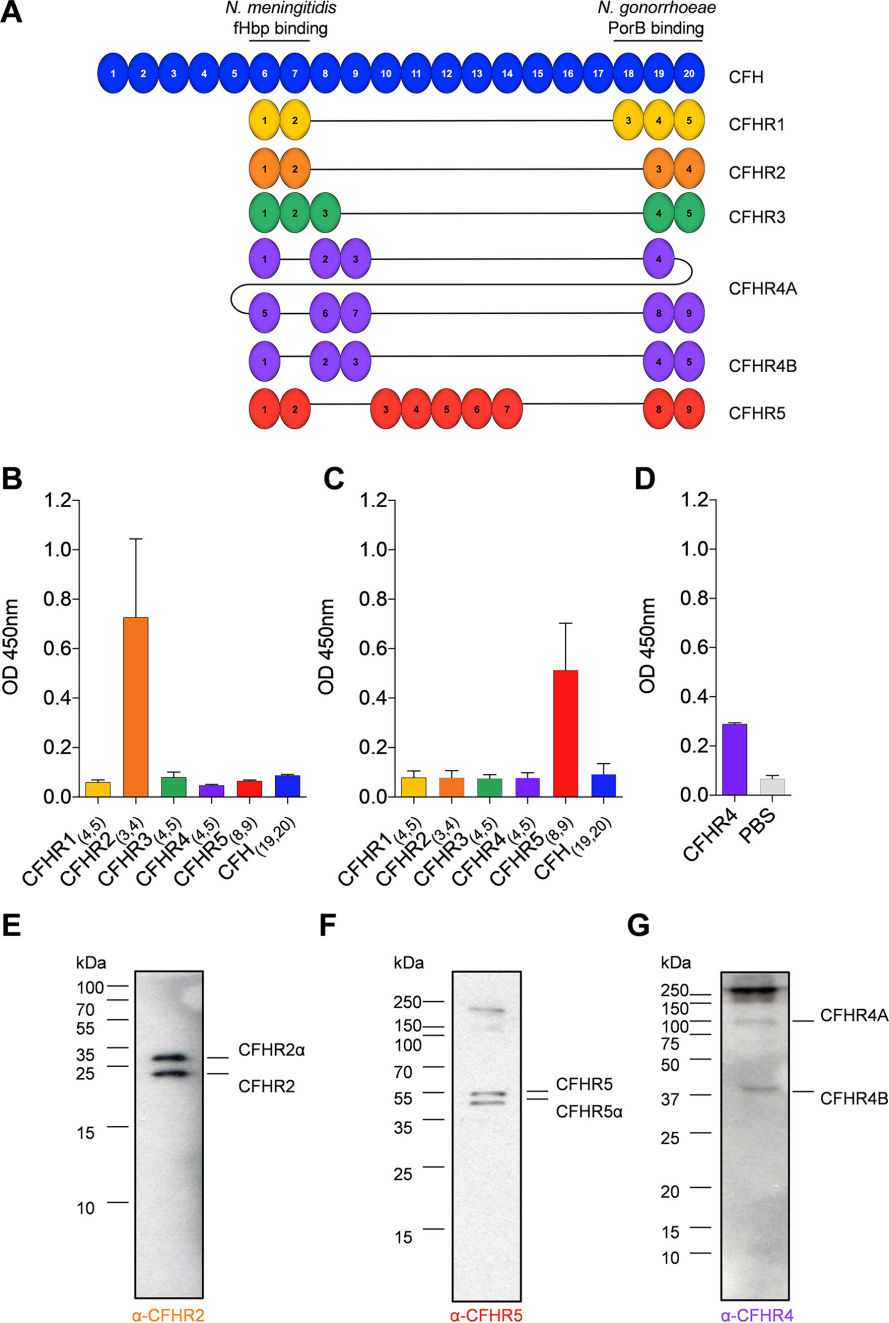
Characterization of anti-CFHR MAbs. (A) Complement control protein (CCP) domains of CFH and CFHRs 1 to 5 are shown with CCPs aligned with domains of CFH that share sequence homology. CFH CCPs that bind to *Neisseria* spp. are indicated with their known receptors. Detection of (B, E) CFHR2, (C and F) CFHR5, and (D and G) CFHR4 by MAbs; HSL-2, HSL-5, and HSL-4, respectively, generated against either the C-terminal pair of CCP domains (CFHR2_(3, 4)_ and CFHR5_(8, 9)_) or peptide (CFHR4). MAbs were screened by ELISA for specificity against the C-terminal pair of CFHR CCP domains (B and C) or the peptide used HSL-4 MAb generation. (D) S.D. of independent assays (*n* = 2) is indicated. (F and G) Western blots of NHS probed with each of the generated CFHR MAbs to determine specificity.

Aside from CFH, five complement factor H-related proteins (CFHR1-5) are also composed of CCPs and share significant structural and sequence similarities with CFH. Of note, all CFHRs lack CCPs related to the complement regulatory domains of CFH_(1-4)_ (Fig. 1A). However, CFHRs 1_(4,5)_, 2_(3,4)_, and 5_(8,9)_ share >40% amino acid similarity with CFH_(19,20)_ (7). The homology of the CFHRs to the surface recognition domains of CFH_(19-20)_ enables these proteins to bind similar or the same molecules on surfaces. However, as the CFHRs lack CCPs required for complement regulatory activity (CFH_[1-4]_), there is accumulating evidence that CFHRs can modulate complement activity (7, 8), with genetic studies indicating CFHRs’ importance in disease. For example, the loss of *CFHR3-CFHR1* or *CFHR1-CFHR4*, or the expression of CFHR1 variants, including CFHR:CFH hybrids can result in atypical hemolytic Uraemic syndrome (aHUS) (reviewed in reference (9)). Several CFHRs, including CFHR2, 3 and 5 can antagonize the regulatory activity of CFH by competing for binding sites on activating surfaces (7). CFHR5 can also activate complement by recruiting other complement proteins such as properdin into a heteromeric complex (10, 11). Despite this, there are still relatively few studies on CFHRs, partly because of the paucity of reagents that can discriminate between them.

Many bacterial pathogens have evolved to recruit CFH *via* CFH_(6,7)_ or instead of and CFH_(19,20)_ to subvert the complement system (12). However, little is known about the interactions between bacteria and CFHRs. Due to the extensive sequence identity of CFHR1_(3-5)_ with CFH_(18-20)_ (Fig. 1A), CFHR1 can bind to several bacteria, including *Borrelia*, Pseudomonas aeruginosa, Staphylococcus aureus, and Neisseria gonorrhoeae (13–16). *Borrelia* also binds CFHRs 2 and 5 (13). Furthermore, CFHR3 can compete with CFH for binding to the surface of Neisseria meningitidis (17), highlighting the potential role of CFHRs during bacterial infections.

N. meningitidis and N. gonorrhoeae are closely related human-adapted pathogens which recruit CFH to subvert complement activation. N. meningitidis is a leading cause of sepsis and bacterial meningitis, whereas N. gonorrhoeae is responsible for the sexually transmitted infection, gonorrhea (18). N. meningitidis recruits CFH *via* a surface lipoprotein, factor H binding protein (fHbp) (19), which binds to CFH_(6,7)_ with nanomolar affinity (17). In the absence of fHbp and capsule, meningococcal NspA and PorB can associate with CFH (20, 21). Gonococcal fHbp is not surface-expressed and does not bind CFH (22). Instead, N. gonorrhoeae binds CFH_(18-20)_ *via* PorB (23), an integral outer membrane porin with eight surface exposed loops (L1-L8). Gonococci express one class of PorB, P.IA, or P.IB, which share >80% nucleotide sequence identity (24, 25). Different loops of P.IA and P.IB are responsible for CFH binding, with L5 of P.IA and L3-7 of P.IB involved in binding CFH (26, 27). Furthermore, the addition of sialic acid to bacterial lipopolysaccharide (LPS) by the α-2,3-sialyltransferase, Lst (28), is required for CFH binding to gonococcal P.IB but not P.IA (26, 27). Little is known about CFHR binding by pathogenic *Neisseria*, although meningococcal fHbp binds both CFHR3 and CFH (17). However, nothing is known about the interactions of CFHR2, 4, and 5 with pathogenic *Neisseria*.

Here, we describe the generation and characterization of monoclonal antibodies (MAbs) that specifically bind to CFHR2, 4, and 5. Using these MAbs, we revealed that CFHR5, but not CFHR2 or 4, bound to PorB from both N. meningitidis and N. gonorrhoeae only when the LPS was sialylated. We identified the loops of N. gonorrhoeae PorB that were responsible for interactions with CFHR5 and showed that there were similarities with the regions of PorB that engaged CFH. Furthermore, we demonstrated that surface-bound CFHR5 enhanced N. gonorrhoeae susceptibility to human complement.

(Work has been previously presented at American Society for Microbiology Microbe 2020 conference by W.X.Y. Title: CFHR5 binds to *Neisseria gonorrhoeae*. Authors: W.X.Y., H.L. and C.M.T.)

## RESULTS

### Generation of specific MAbs against CFHRs.

Understanding the function of CFHRs has been hampered by the lack of specific reagents, with many antibodies against CFH and CFHRs cross-reacting with other family members because they share considerable amino acid identity (Fig. 1A). We previously isolated a specific anti-CFHR3 MAb, HSL-1 (17), while OX-24 is a MAb which specifically recognizes CFH_(5)_ (29). However, few specific reagents have been generated against other CFHRs (30–32). We generated anti-CFHR 2, 4, and 5 MAbs by standard hybridoma technology (17) using recombinant CFHR2_(3,4)_, CFHR5_(8,9)_, or a synthetic peptide from CFHR4_(3)_ as antigens. Candidate anti-CFHR2 and anti-CFHR5 MAbs were screened by an enzyme-linked immunosorbent assay (ELISA) for cross-reactivity to CFH and other CFHRs using the two C-terminal CCPs of CFH and CFHRs 1 to 5. The results identified specific MAbs against CFHR2 and CFHR5, designated HSL-2 and HSL-5, respectively (Fig. 1B and C). A MAb against CFHR4, designated HSL-4, recognized the immunizing CFHR4 peptide by an ELISA (Fig. 1D). Furthermore, the specificity of MAbs HSL-2, -5, and -4 was assessed by Western blotting of normal human serum (NHS) to demonstrate the molecular weight of proteins detected, identify cross-reactivity with other serum proteins, and determine if MAbs recognize known posttranslational modification*s, e.g.*, glycosylation of CFHRs (8) (Fig. 1E to G). Both HSL-2 and HSL-5 recognized two bands in NHS consistent with the molecular masses of differentially glycosylated forms of CFHR2 and CFHR5 that have previously been described (8) (Fig. 1E and F). *CFHR4* encodes two splice variants, CFHR4A, and CFHR4B, with approximate molecular masses of 86 and 42 kDa, respectively (33), both of which were recognized by HSL-4 (Fig. 1A and G). Importantly, the three MAbs specifically recognized the immunizing CFHR and no other protein in NHS by ELISA and Western blot (Fig. 1B to G).

### CFHR5 bound to PorB from N. meningitidis.

**FIG 2:**
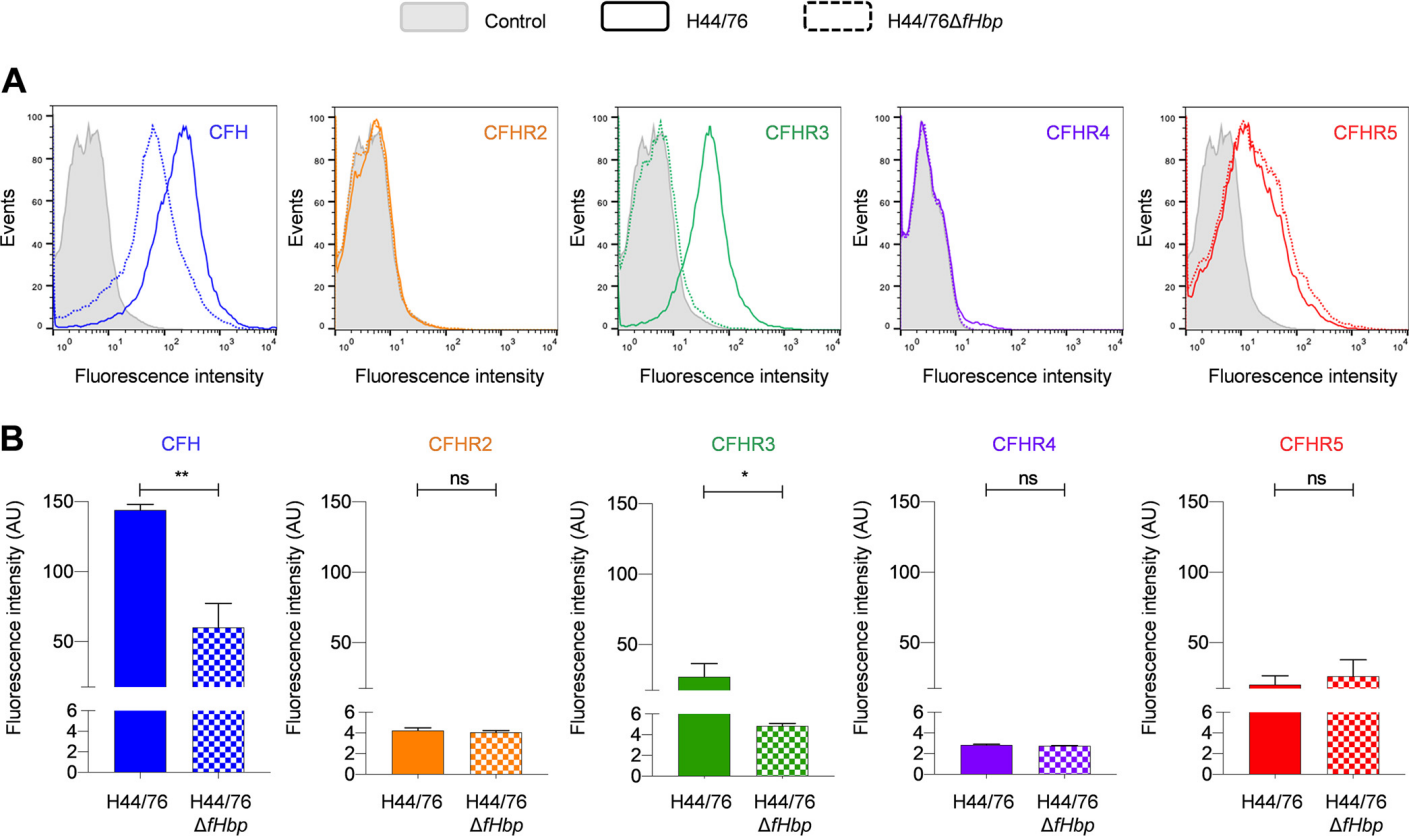
N. meningitidis bound CFHR5 independent of fHbp. Flow cytometry was used to detect the binding of CFH and CFHRs to N. meningitidis H44/76 and H44/76Δ*fHbp*. CFH (blue), CFHR2 (orange), CFHR3 (green), CFHR4 (purple) and CFHR5 (red) in NHS using specific MAbs. Bacteria incubated with no primary antibody (gray trace) were used as a control. (A) Representative histograms and (B) comparison of geometric mean fluorescence intensity and S.D. of independent assays (*n* ≥ 3) are as indicated. Unpaired *t* tests were used to compare the fluorescence intensity of CFHR and CFH MAb binding between wild-type N. meningitidis H44/76 and H44/76Δ*fHbp*. ns, *P* ≥ 0.05; *, *P ≤ *0.05; **, *P ≤ *0.01.

With these MAbs, we next determined whether CFHR2, 4, or 5 bind to N. meningitidis. Because both CFH and CFHR3 bind N. meningitidis *via* fHbp (17, 19), we wanted to establish if any potential interaction was dependent on fHbp. Flow cytometry demonstrated that N. meningitidis bound CFH and CFHR3 in an fHbp-dependent manner as previously (17) (Fig. 2, binding of CFH and CFHR3 to N. meningitidis H44/76Δ*fHbp P* = 0.0136 and 0.0012, respectively, compared with wild-type bacteria, unpaired two-tailed *t* test). In contrast, no significant binding of CFHR2 or CFHR4 was detected to N. meningitidis in the presence/absence of fHbp (Fig. 2; *P* = 0.3473 and *P* = 0.0935 using an unpaired two-tailed *t* test). However, we found that CFHR5 bound to N. meningitidis H44/76 independent of fHbp (wild-type strain versus H44/76Δ*fHbp*, *P* = 0.4799, unpaired two-tailed *t* test, Fig. 2).

**FIG 3:**
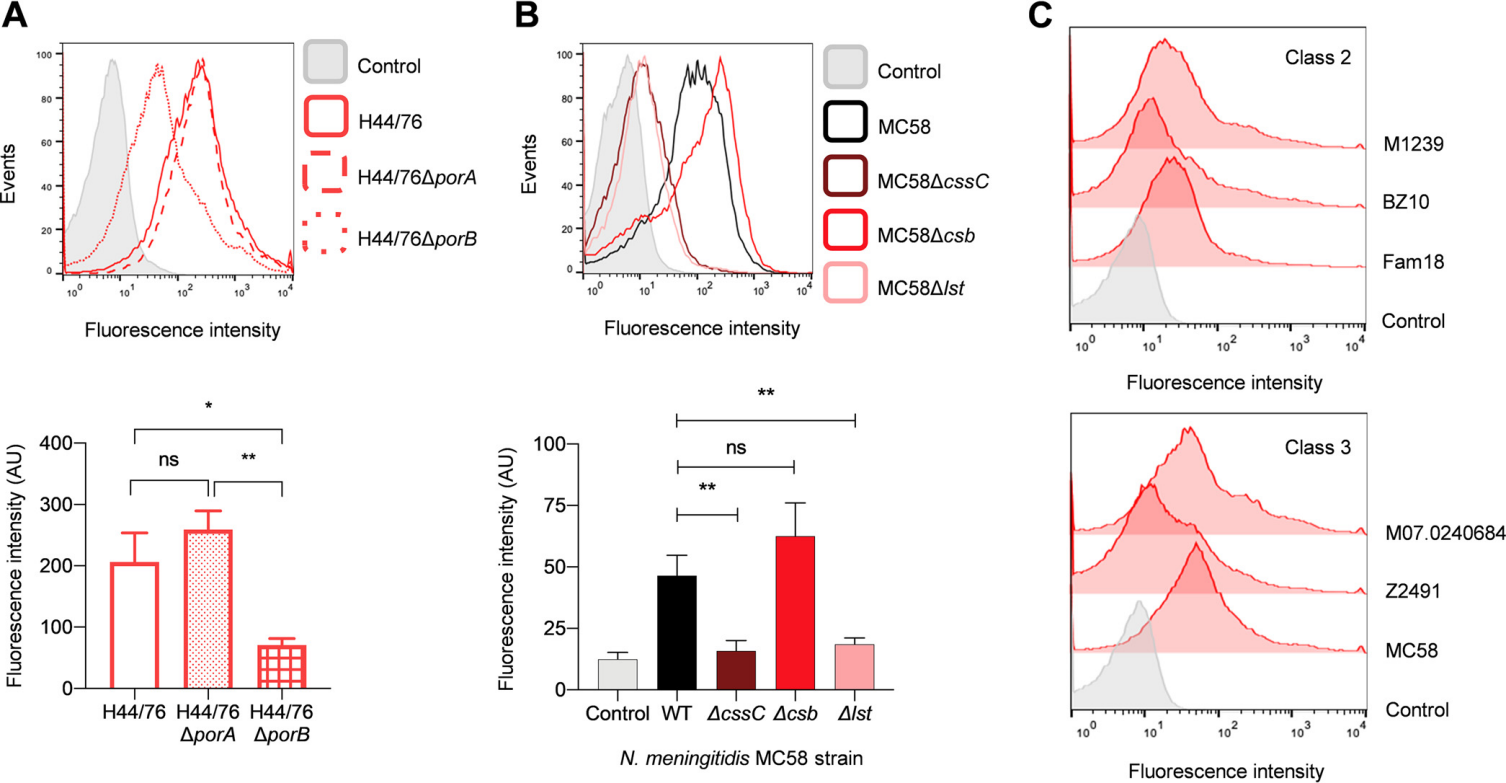
CFHR5 bound to N. meningitidis PorB. (A) Flow cytometry analysis demonstrates the binding of CFHR5 to wild-type N. meningitidis H44/76 (solid line), H44/76Δ*porA* (dashed line), and H44/76Δ*porB* (dotted line); representative histograms are shown. (B) Flow cytometry analysis of CFHR5 binding to wild-type N. meningitidis MC58 (black), MC58Δ*cssC* (dark red), MC58Δ*csb* (red), and MC58Δ*lst* (light red); representative histograms are shown. Comparison of geometric mean fluorescence intensity and S.D. of independent assays (*n* ≥ 3) are indicated. Unpaired *t* test and one-way ANOVA method of multiple comparisons were used to compare the fluorescence intensity of CFHR5 binding and PorA/PorB levels versus control samples lacking the primary antibody (gray); ns, *P* ≥ 0.05; *, *P ≤ *0.05; **, *P ≤ *0.01. (C) CFHR5 bound N. meningitidis expressing class 2 and class 3 PorB by flow cytometry.

N. gonorrhoeae binds CFH to a region of external loop 5 of Por.IB. N. meningitidis expresses two outer membrane porins, PorA and PorB which share 40 to 50% and 60 to 70% amino acid identity, respectively, with gonococcal PorB (34, 35). Furthermore, CFHR5_(8,9)_ shares 40 to 70% similarity with CFH_(19,20)_ (7), the latter bound to meningococcal PorB (20). Therefore, we hypothesized that CFHR5 might bind to either meningococcal PorA or PorB. We assessed CFHR5 binding to isogenic Δ*porA* or Δ*porB* mutants using NHS as the source of CFHR5. Of note, the Δ*porB* mutant exhibited significantly reduced CFHR5 binding compared to the wild-type strain (*P* = 0.0485, one-way analysis of variance [ANOVA]; Fig. 3A), indicating that PorB is a receptor for CFHR5 on the meningococcus. There was a nonsignificant increase in CFHR5 binding to H44/76Δ*porA* (*P* = 0.4011, one-way ANOVA), consistent with the nonsignificant increase in PorB expression by the Δ*porA* strain (*P* = 0.5292, one-way ANOVA; Fig. S1 in Supplemental File 1).

Because CFH binding to gonococcal PorB can be modulated by the addition of sialic acid to LPS (26), we next determined the influence of structures containing sialic acid on CFHR5 binding to N. meningitidis MC58, which expresses a polysialic acid capsule and endogenously sialylates its LPS (36). We examined CFHR5 binding to strains that were defective for LPS sialylation (MC58Δ*lst*), capsule biosynthesis (MC58Δ*csb*), or both (MC58Δ*cssC*, which is unable to synthesize sialic acid (37)). While there was no significant difference in CFHR5 binding to MC58Δ*csb* compared with the wild-type strain (*P* = 0.0792, one-way ANOVA), there was no detectable CFHR5 binding to MC58Δ*cssC* or MC58Δ*lst* (Fig. 3B; *P* = 0.0019 and *P* = 0.0037, respectively, compared to wild-type, one-way ANOVA). Therefore, CFHR5 binding to N. meningitidis PorB is dependent on LPS sialylation.

N. meningitidis expresses one of two classes of PorB, PorB2 or PorB3, encoded by different alleles at the *porB* locus (34). Therefore, we examined whether PorB belonging to different classes exhibited differences in CFHR5 binding. Flow cytometry of CFHR5 binding to a range of isolates indicated that both classes of meningococcal PorB mediated CFHR5 binding (Fig. 3C).

### N. gonorrhoeae bound CFHR5.

**FIG 4:**
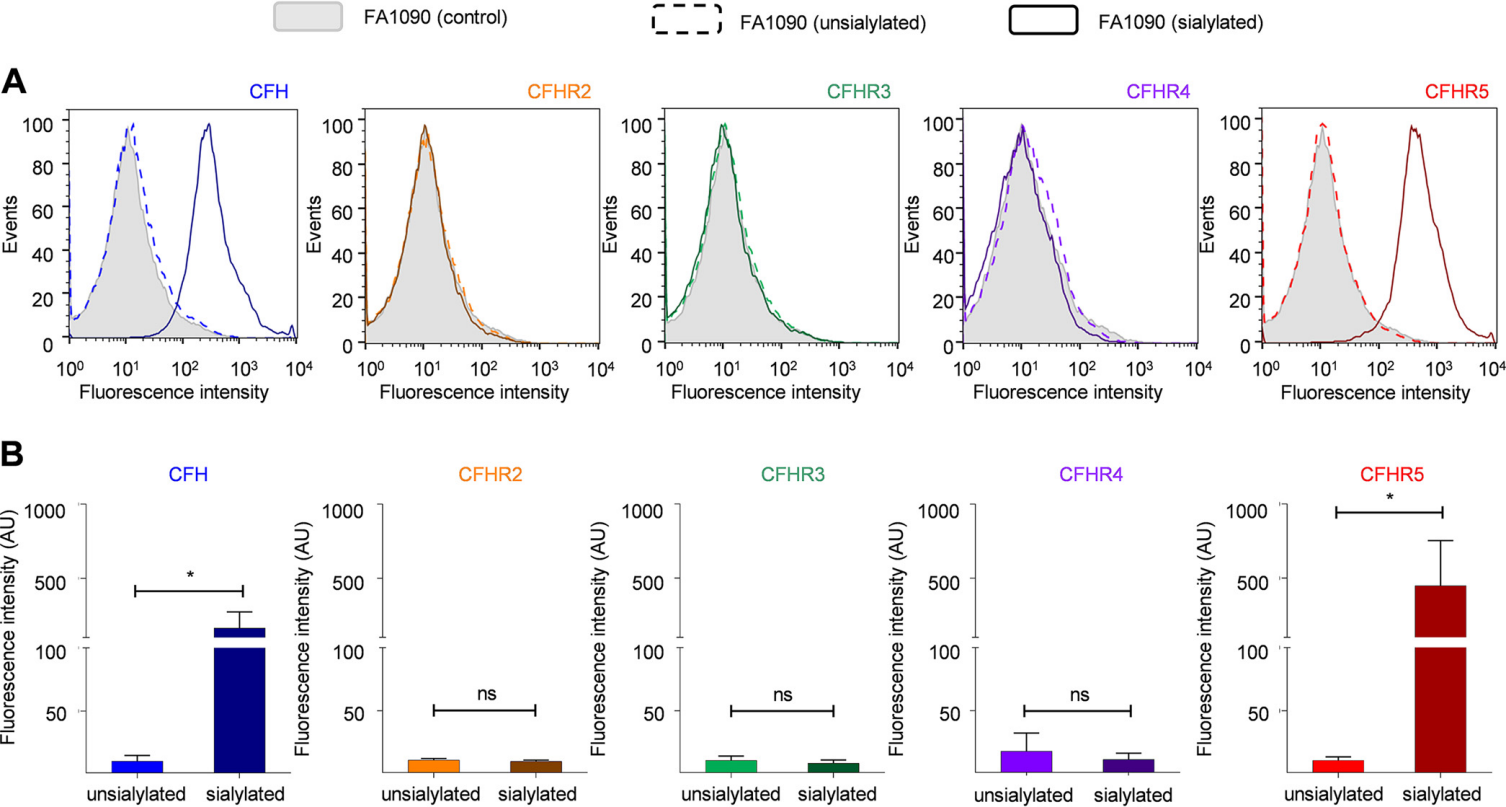
N. gonorrhoeae FA1090 bound CFHR5. Flow cytometry analysis of N. gonorrhoeae FA1090 incubated in NHS as the source of CFH (blue) CFHR2 (orange), CFHR3 (green), CFHR4 (purple), and CFHR5 (red). CFH-CFHR binding was detected with specific MAbs against CFH and CFHRs. (A) Representative histograms show the binding of CFH and CFHRs to unsialylated (dotted lines) and sialylated bacteria (solid lines). Bacteria incubated with no primary antibody were used as negative controls (filled gray). (B) Geometric mean fluorescence intensity and S.D. of independent flow cytometry experiments (*n* ≥ 3) are also indicated. Values were analyzed with an unpaired *t* test. ns, *P* ≥ 0.05; *, *P ≤ *0.05.

We next examined whether N. gonorrhoeae bound to CFHRs 2 to 5. We assessed CFHR binding to N. gonorrhoeae FA1090 grown with or without cytidine-59-monophosphate-N-acetylneuraminic acid (CMP-NANA) as N. gonorrhoeae only sialylates its LPS when provided with an exogenous source of sialic acid. CFHRs/CFH binding was detected by flow cytometry using relevant MAbs. The results demonstrated that N. gonorrhoeae bound CFH and CFHR5, but not CFHR2, 3, or 4 (Fig. 4A) under the conditions tested. The binding of both CFH and CFHR5 was dependent on the presence of sialic acid during growth (*P* = 0.0288 and *P* = 0.0281, respectively, unpaired two-tailed *t* test; Fig. 4B). These findings were confirmed using purified CFH and recombinant CFHR5 (*P ≤ *0.0001 and *P* = 0.0032, respectively, compared to bacteria grown without CMP-NANA, using unpaired two-tailed *t* test; Fig. S2 in Supplemental File 1), which demonstrated that no binding of either protein in the absence of LPS sialylation.

### Loops 1 and 6 of gonococcal PorB showed differential binding of CFH and CFHR5.

**FIG 5:**
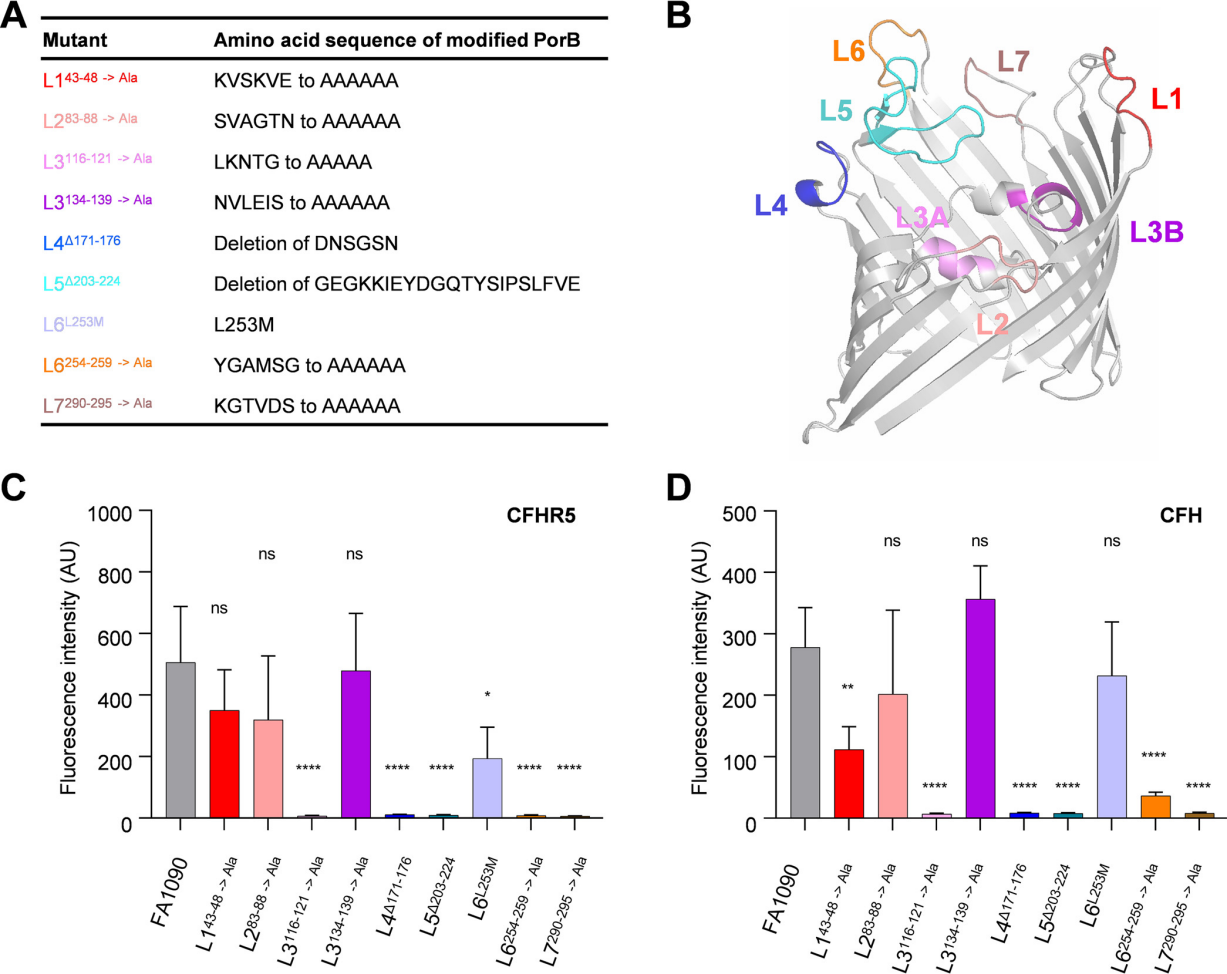
N. gonorrhoeae FA1090 with mutations in loops 1 and 6 have differential ability to bind CFH and CFHR5. (A) Table of N. gonorrhoeae FA1090 derivatives with modified P.IB (26). (B) Predicted structure of N. gonorrhoeae P.IB PorB modeled in Phyre V 2.0 using meningococcal P.IB PDB 3VZT (47) as the template; image generated in PyMol. The sites of modifications in P.IB are indicated. (C) Binding CFHR5 to N. gonorrhoeae FA1090 was determined by flow cytometry. L3^116-121→Ala,^ L4^Δ171-176^, L5^Δ203-224^, L6^254-259→Ala^, and L7^290-295→Ala^ resulted in undetectable levels of CFHR5 binding. L6^L253M^ showed significantly decreased CFHR5 binding. (D) Binding of serum-derived CFH to FA1090 P.IB loop mutants was determined by flow cytometry. L3^116-121→Ala^, L4^Δ171-176^, L5^Δ203-224^, L7^290-295→Ala^ resulted in a loss of CFH binding. L1^43-48→Ala^ and L6^254-259→Ala^ showed significantly decreased CFH binding. Results are shown as geometric mean fluorescence intensity with S.D. (*n* ≥ 3 independent assays). Significance was analyzed using one-way ANOVA, with wild-type N. gonorrhoeae FA1090 as the control. ns, *P* ≥ 0.05; *, *P ≤ *0.05; **, *P* ≤ 0.01; ****, *P ≤ *0.0001.

We hypothesized that gonococcal PorB might also be the target for CFHR5 binding, similar to the meningococcus. N. gonorrhoeae expresses one of two classes of PorB, P.IB, and P.IA (34, 38). FA1090 expresses P.IB PorB. PorB is essential in N. gonorrhoeae (39) therefore, we examined CFHR5 binding to strains with modified P.IB surface loops that have previously been used to characterize CFH binding (Table 1 and Fig. 5A) (26). We also substituted leucine at position 253 with methionine in loop 6 as this residue is involved in binding the negative complement regulator C4bp (26). Strains with modified PorB loops were designated Ln^xxx^, with Ln representing the PorB loop number, and xxx representing the amino acid(s) substituted (→) or deleted (Δ) (Table 1 and Fig. 5A). Modification of PorB loops did not affect PorB expression or growth of bacteria (Fig. S3 in Supplemental File 1). Bacteria were incubated with NHS as the source of CFH and CFHR5 which were detected with MAbs OX-24 or HSL-5, respectively. Strains L3^116-121→Ala^, L4^Δ171-176^, L5^Δ203-224^, and L7^290-295→Ala^ exhibited loss of both CFH and CFHR5 binding (*P ≤ *0.0001 compared to wild-type FA1090, one-way ANOVA, Fig. 5C and D). Of note, while L6^254-259→Ala^ showed a significant decrease in CFH binding compared with wild-type bacteria, CFHR5 binding was not detected (*P ≤ *0.0001, one-way ANOVA). In contrast, there was a significant decrease in CFH binding to strain L1^43-48→Ala^ (*P* = 0.0019, one-way ANOVA), while CFHR5 binding was unchanged. Interestingly, strain L6^L253M^ showed a significant decrease in CFHR5 binding (*P* = 0.0154 using one-way ANOVA), while CFH binding was unaffected. L2^83-88→Ala^ and L3^134-139→Ala^ exhibited no change in CFH (*P* = 0.3931 and *P* = 0.3577, respectively, one-way ANOVA) or CFHR5 binding compared with the wild-type strain (*P* = 0.3301 and *P* = 0.9997, respectively, one-way ANOVA) (Fig. 5C and D). Overall, the results indicated that, while several PorB modifications had similar effects on CFHR5 and CFH binding, modification of L1 affected the binding of CFH more than CFHR5, while modification of L6 reduced CFHR5 binding disproportionately compared with CFH.

**TABLE 1:** *Neisseria* species isolates^*a*^

Strain	Year	Country	cc	ST	Strain type	Reference
Neisseria meningitidis						
H44/76	1976	Norway	32	32	B:15:P1.7,16	53
H44/76Δ*fHbp*	-	-	-	-	-	22
H44/76Δ*porA*	-	-	-	-	-	54
H44/76Δ*porB*	-	-	-	-	-	This study
MC58	1985	UK	32	74	B:15:P1.7,16b	55
MC58Δ*cssC*	-	-	-	-	-	37
MC58Δ*csb*	-	-	-	-	-	37
MC58Δ*lst*	-	-	-	-	-	37
M1239	1994	USA	41/44	437	B:14:P1.23,14	56
BZ10	1967	Netherlands	8	8	B: P1.5-1,2-2	57
Fam18	1983	USA	11	11	C:2a:P1.5,2	58
Z2491	1983	Africa	4	4	A: P1.7,13-1	59
M07.0240684	2007	UK	23		Y	60
Neisseria gonorrhoeae						
FA1090 G4	1980	USA				61
L1^43-48→Ala^	-	-				26
L2^83-88→Ala^	-	-				26
L3^116-121→Ala^	-	-				26
L3^134-139→Ala^	-	-				26
L4^Δ171-176^	-	-				26
L5^Δ203-224^	-	-				26
L6^L253M^	-	-				26
L6^254-259→Ala^	-	-				26
L7^290-295→Ala^	-	-				26
NG102	2012	UK				22
NG104	2012	UK				22
NG118	2012	UK				22
65737	2014	Kenya				62
60755	2013	Kenya				62
55496	2012	Kenya				62

a -, the information is not available due to it being a strain generated in the study.

### CFHR5 bound a range of gonococcal isolates.

**FIG 6:**
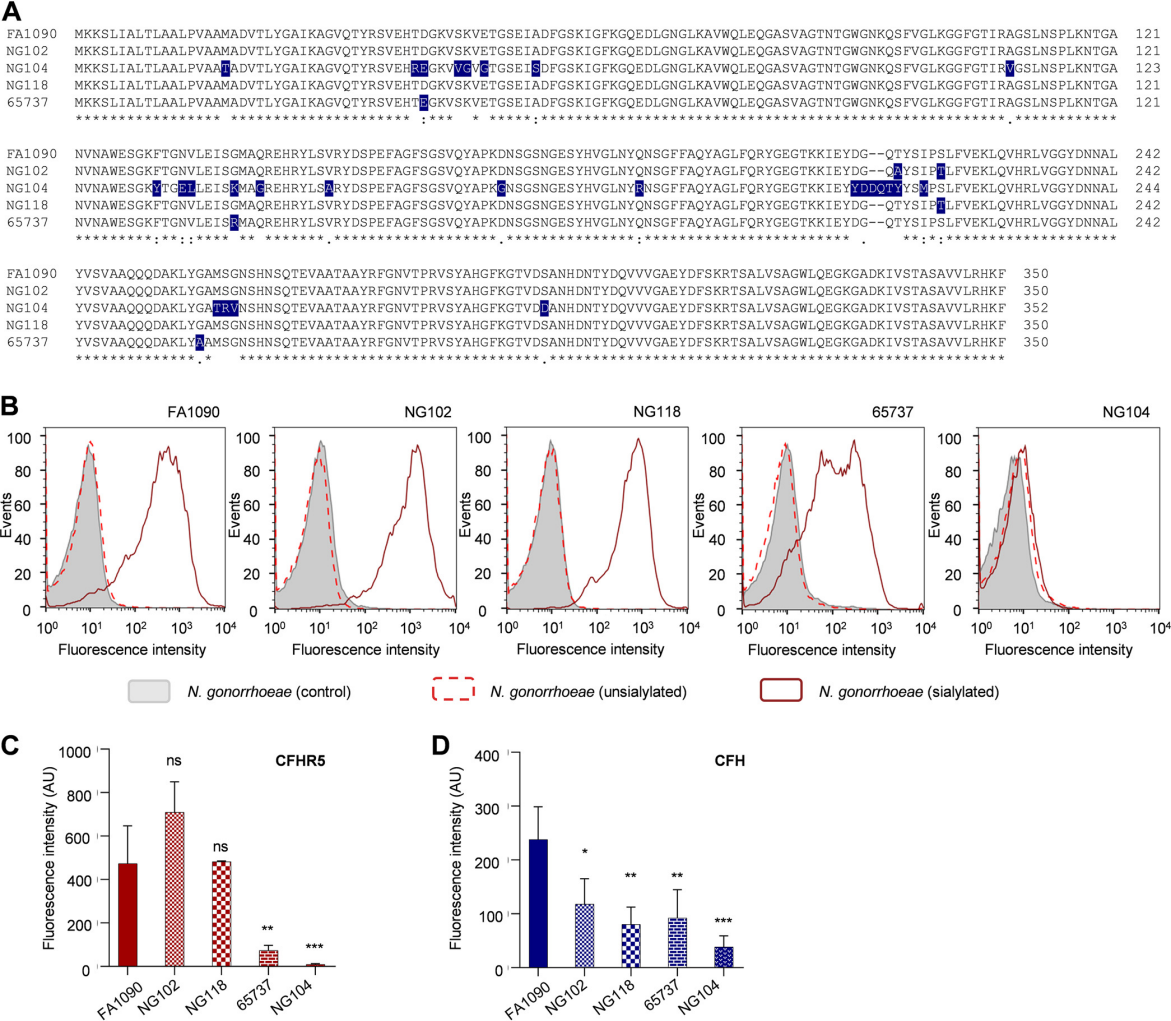
Clinical isolates of N. gonorrhoeae bound CFHR5, highlighting the importance of P.IB loops 5 and 6. (A) Alignment of P.IB amino acid sequences of N. gonorrhoeae FA1090, NG102, NG104, NG118, and 65737, generated by Clustal Omega. Amino acids found in the minority of isolates are highlighted. (B) Representative histograms of CFHR5 binding to clinical isolates NG102, NG118, and 65737. Isolates still exhibited CFHR5 binding which was dependent on LPS sialyation (solid line). NG104, which had several substitutions in P.IB, did not bind CFHR5. CFHR5 binding (C) and CFH binding (D) are shown as geometric mean fluorescence intensity with S.D. (*n* ≥ 3 independent assays). 65737 and NG104 showed reduced CFHR5 binding compared with N. gonorrhoeae FA1090; all clinical isolates showed reduced CFH binding compared to FA1090. Significance was analyzed with an *unpaired t test*, with wild-type N. gonorrhoeae FA1090 as control. ns, *P* ≥ 0.05; *, *P ≤ *0.05; **, *P* ≤ 0.01; ***, *P* ≤ 0.001.

Next, we further interrogated which PorB loops are important for CFHR5 and CFH binding using clinical isolates with amino acid substitutions in PorB compared to FA1090 P.1B (Fig. 6A). Whole-genome sequences were interrogated using the PubMLST *Neisseria* BIGSdb database (https://pubmlst.org/organisms/neisseria-spp) (40). The results demonstrated that N. gonorrhoeae NG104, which has numerous substitutions in loops 1, 3, and 6 and an insertion of two amino acids in loop 5, did not bind CFHR5 (Fig. 6B and C) and exhibited reduced CFH binding (Fig. 6D; *P* = 0.0003, one-way ANOVA). Analysis of strain 65737, which has amino acid substitutions in loops 1, 3, and 6, also showed a reduction of CFHR5 and CFH binding compared with FA1090 (Fig. 6B to D; *P* = 0.0017 and *P* = 0.0039, respectively, one-way ANOVA). The role of loop 5 was examined using isolates NG102 and NG106 which have one or two amino acid substitutions in this loop compared with N. gonorrhoeae FA1090. Both these strains bind CFHR5 to a similar extent but had significantly reduced CFH binding (*P* = 0.0155 and *P* = 0.0021 versus FA1090, respectively, one-way ANOVA) (Fig. 6B to D). This suggested that specific residues on loop 5 may exhibit differential binding to CFH and CFHR5. Of note, two clinical isolates expressing PorB and P.IA did not bind CFHR5 (Fig. S4 in Supplemental File 1).

### Binding of CFHR5 to N. gonorrhoeae enhanced complement activation.

**FIG 7:**
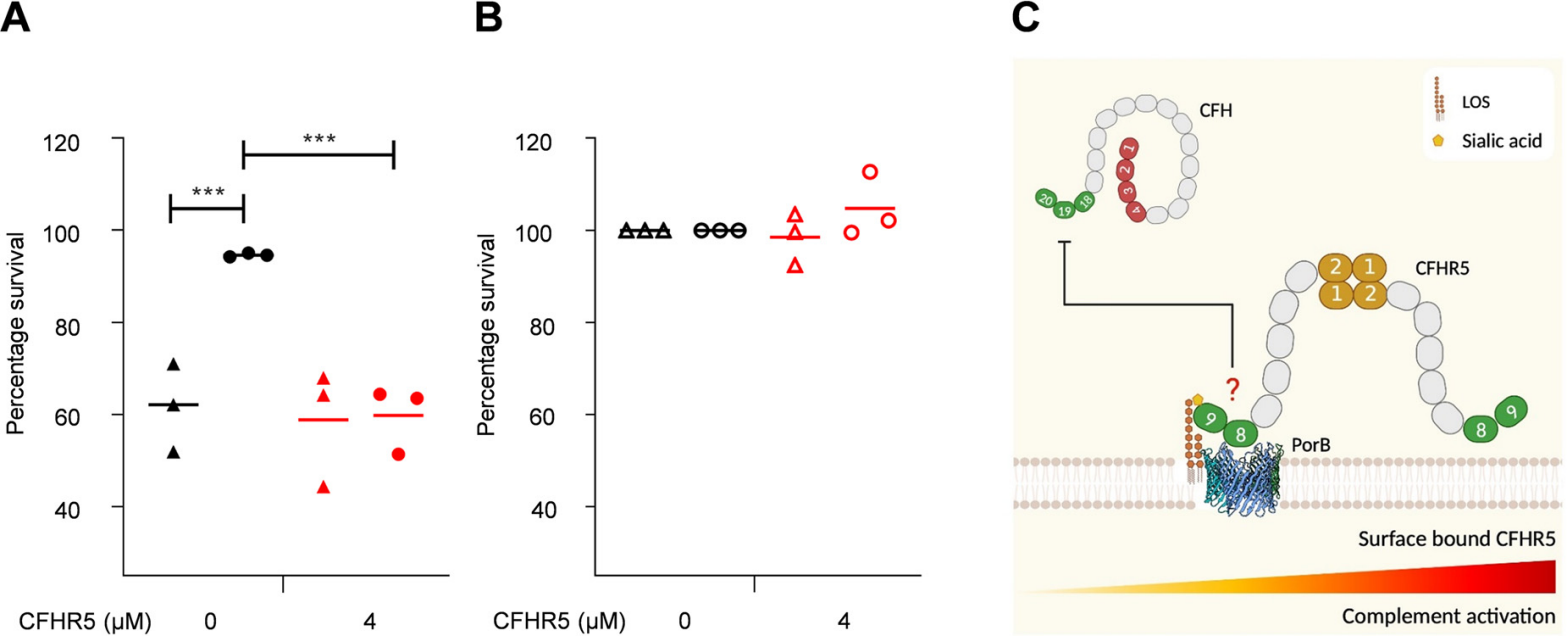
CFHR5 increases the susceptibility of sialylated N. gonorrhoeae FA1090 to complement-mediated lysis. Sensitivity of sialylated (circles) and unsialylated (triangles) N. gonorrhoeae FA1090 to complement-mediated lysis after preincubation with 4 μM CFHR5 (red) or PBS (black). Bacteria were incubated in HI-hComplement (A) or hComplement (B). Data are presented as percentage survival relative to bacteria incubated in HI-hComplement without added CFHR5. Statistical significance was tested using the two-way ANOVA multiple comparisons as implemented in GraphPad Prism v.9.0 (GraphPad Software Inc.) to compare means ±S.D. using a *P* < 0.05 cutoff for significance. ***P < 0.001; ns *P* > 0.05. (C) Model of how CFHR5 influences N. gonorrhoeae susceptibility to complement-mediated lysis. CCPs 18 to 20 of CFH compete with CFHR5, potentially by the homologous CCPs 8 to 9 to bind to gonococcal P.1B in the presence of sialylated LPS. Increased levels of surface-bound CFHR5 which lack the complement regulatory domains of CFH_(1-4)_ allow complement activation and amplification to proceed unhindered consequently resulting in increased bacterial lysis. Created with BioRender.com; PorB structure, PDB ID 4AUI (46).

To examine the biological consequences of N. gonorrhoeae binding CFHR5, strain FA1090 was preincubated with 4 μM exogenous recombinant CFHR5 before the addition of antibody-depleted human complement (hComplement). Preincubation of sialylated FA1090 with CFHR5 resulted in significantly reduced survival of FA1090 (two-way ANOVA *P* = 0.0005) compared to bacteria with no CFHR5 (Fig. 7A). In contrast, CFHR5 did not affect bacterial survival when LPS was not sialylated (two-way ANOVA *P* = 0.9995) (Fig. 7A). Furthermore, the addition of CFHR5 did not affect bacterial survival of sialylated or unsialylated FA1090 in the presence of Heat-inactivated (HI)-hComplement (two-way ANOVA, *P* = 0.9864 and >0.9999, respectively) (Fig. 7B). Of note, unsialylated FA1090 was significantly more susceptible to complement-mediated lysis than sialylated bacteria as expected (two-way ANOVA, *P* = 0.0009) (Fig. 7A).

## DISCUSSION

Complement is a crucial aspect of the innate immune system and defense against invading pathogens. This is highlighted by the susceptibility of individuals with complement defects to infectious diseases, and the array of complement evasion mechanisms exhibited by pathogenic bacteria (reviewed in reference (2)). Recruitment of CFH, the major negative regulator of the AP, is exploited by pathogenic *Neisseria* to subvert complement-mediated attack. However, unlike CFH, little is known about CFHRs binding to bacterial pathogens, with the study of CFHRs hampered by the lack of specific reagents, although recently MAbs that recognize CFHR2, 4, and 5 have been described and used to measure the respective CFHR levels in serum (30, 31, 41). Here, we generated MAbs which specifically recognize CFHR2, 4, and 5 and utilized them to detect the binding of these proteins to N. meningitidis and N. gonorrhoeae.

Even though CFH and the CFHRs share high sequence and structural similarity, we successfully generated specific MAbs using recombinant CFHR2_(3,4)_, CFHR5_(8,9)_, and a linear peptide of CFHR4_(3)_, which recognize different glycosylated forms of these proteins in serum. This indicates that the MAbs recognize epitopes distinct from posttranslational modification sites (8). Furthermore, our anti-CFHR4 MAb recognizes both splice variants of CFHR4 in NHS, contrary to previous studies which suggest that only a single variant is present in serum. This discrepancy could be explained by the variable circulating levels of CFHR4 (31). While there are limited data on the function of CFHRs, genetic studies have highlighted their contribution to complement-mediated diseases and in particular meningococcal infection (42–44). Therefore, these MAbs will help assess CFHR levels during health and disease, and the interaction of CFHRs with pathogenic microbes as exemplified here.

Here, we used serum-derived CFH and CFHRs 2 to 5 to detect binding to N. meningitidis and N. gonorrhoeae. This ensures that the proteins are posttranslationally modified and correctly folded. As previously found (17, 19), we demonstrated that CFHR3 was bound to N. meningitidis in an fHbp-dependent manner. Furthermore, the minor residual CFH binding to the Δ*fhbp* mutant can be attributed to its interaction with NspA and PorB2, as previously described (21, 45). The gonococcal homolog of fHbp is not surface exposed (22), explaining why we detected no CFHR3 binding to N. gonorrhoeae. N. gonorrhoeae has been shown to bind purified CFHR1 (16). However, we could not confirm this using serum CFHR1. Of the other CFHRs, we only detected CFHR5 binding to the meningococcus and the gonococcus. However, we could not exclude the possibility that pathogenic *Neisseria* also bound CFHRs 2 and 4. Serum levels of CFHR4, CFHR2, and heterodimers of CFHR1 and 2 are relatively low (2.55 ± 1.46, 0.7 ± 0.4, and 5.8 ± 2.4 μg/mL, respectively) (30, 32) which might limit their detection in our assays (30, 32, 41). Therefore, the ability of N. gonorrhoeae and N. meningitidis to bind CFHR2 and 4 should be investigated using purified proteins.

PorB is an outer membrane porin that forms a 16-stranded β-barrel with eight outer membrane loops and assembles into trimers (46, 47). Both N. meningitidis and N. gonorrhoeae strains express one of two mutually exclusive PorB classes (PorB2 and PorB3, and P.IA and P.IB, respectively) (34). In N. meningitidis, isolates expressing either class 2 or 3 PorB bind CFHR5. Therefore, CFHR5 might bind to the nonvariable, surface-exposed L2, 3, 4, or 8. In contrast, CFH has been hypothesized to bind to variable loops 1,5,6, and 7 (20). In N. gonorrhoeae, both P.IA and P.IB share 65 to 80% amino acid homology (48), with main differences in the surface exposed loops, especially in loop 5. Of note, P.IA strains did not bind CFHR5 (Fig. S4 in Supplemental File 1). CFHR5 similarly bound gonococcal PorB as CFH, although mutational analysis and examination of clinical isolates revealed that the precise sites of CFH and CFHR5 binding on P.IB differ subtly. Our results suggest that modification of L1 affects CFH binding more than CFHR5, while changes in L6 affect CFHR5 binding more than CFH.

Apart from PorB, we demonstrated that LPS sialylation was required for CFHR5 binding to both the meningococcus and gonococcus. It is unknown how LPS sialylation contributes to the recruitment of complement factors by pathogenic *Neisseria*. CFH recognizes negatively charged surfaces on host and pathogen membranes, such as glycosaminoglycans (reviewed in reference (6)). Therefore, the presence of the negatively charged sialic acid group to LPS might act cooperatively with PorB to form a docking site for complement factor binding or affect the conformation of the extracellular loops of PorB and indirectly facilitate CFH and CFHR5 binding.

The binding of CFHR5 to pathogenic *Neisseria* could influence complement regulation by CFH and result in the recruitment of other immunomodulatory molecules, such as properdin and pentraxins (10). Accumulation of properdin could contribute to complement activation at the site of infection, enhancing complement activity on the bacterial surface as properdin is a positive regulator of the AP (10), provoking local proinflammatory responses. Indeed, low CFHR5 levels in an individual with a *CFHR5* frameshift mutation were associated with renal disease development after Streptococcus pneumoniae infection (49). Our data support this hypothesis as the addition of CFHR5 to sialylated N. gonorrhoeae enhanced the complement-mediated lysis of bacteria (Fig. 7A and B). CFHR5 had no detectable effect when added to unsiaylated N. gonorrhoeae, consistent with the finding that the addition of sialic acid to LPS is crucial for complement evasion (50). This is consistent with CFHR5 modulating complement activation, probably by competing with CFH for binding to PorB (Fig. 7C), in a manner analogous to the competition between CFHR3 and CFH for meningococcal fHbp (17). It is likely that bacteria evolved to recruit CFH but may be unable to distinguish the negative complement regulator from the CFHRs due to their high structural and sequence identity. The outcome of binding on complement activation will be determined by the local levels of CFHR5 and CFH along with their relative affinities for their bacterial target. Of note, high CFHR5 levels have been observed in otitis media with effusion which was hypothesized to compete with CFH for binding of C3b enabling rapid enhancement of complement activation (51). Furthermore, fine-tuning complement activation at the site of infection could be achieved by the therapeutic use of CFHRs to combat infectious diseases. Increasing CFHR levels could result in enhanced AP activation and clearance of bacteria by the immune system.

In summary, we have characterized specific MAbs for detecting CFHR2, 4, and 5, which will help in understanding the function of the CFHRs and their interactions with microbes. Using these MAbs, we demonstrated that CFHR5 was bound to the surface of pathogenic *Neisseria* spp. when LPS was sialylated. While N. meningitidis and N. gonorrhoeae employed distinct strategies to recruit CFH, there were remarkable parallels between the mechanisms of CFHR5 recruitment by these pathogens, where both pathogens bind CFHR5 *via* PorB in the presence of sialylation. Specific PorB loops are involved in CFHR5 binding in N. gonorrhoeae, which antagonized the effect of CFH on bacterial survival in the presence of human complement, highlighting the complex interactions between pathogenic *Neisseria* spp. and human complement.

## MATERIALS AND METHODS

### Bacterial strains and growth.

Bacterial strains used in this study are listed in Table 1. N. meningitidis were grown on brain heart infusion (BHI) agar (1.5% [wt/vol], Oxoid) supplemented with 5% Levinthal's base (500 mL defibrinated horse blood autoclaved with 1 L BHI). N. gonorrhoeae was grown in a gonococcal base liquid medium (GCBL) consisting of 1.5% (wt/vol) proteose peptone number 3 (Becton, Dickinson), 0.1% (wt/vol) starch, 0.4% (wt/vol) K_2_HPO_4_, 0.1% (wt/vol) KH_2_PO_4_, 0.5% (wt/vol) NaCl supplemented with 1% Vitox (Oxoid). Agar 1.5% (wt/vol) (Oxoid) was included for solid medium. Cultures were then incubated for 16 to 18 h at 37°C with 5% CO_2_. Escherichia coli was grown on Luria-Bertani agar (Oxoid) overnight at 37°C. For growth assays, measurements of the Optical Density (A_600_) were taken every hour for 4 h. N. gonorrhoeae was sialylated by the addition of 2 μg mL^−1^ of cytidine-5′-monophosphate-N-acetylneuraminic acid (CMP-NANA) to media. For *Neisseria* spp., kanamycin (kan) and erythromycin were added to media at 50 μg mL^−1^ and 2 μg mL^−1^, respectively. Ampicillin (100 μg mL^−1^) was used for Escherichia coli as required.

### Generation of bacterial strains.

N. meningitidis H44/76Δ*porB* was generated by insertional inactivation of *porB* with a cassette encoding kan resistance. Primer pairs porB forward/porBM reverse (Table S1 in Supplemental File 1 shows all primers), along with porBM forward/porB reverse were designed to amplify *porB* and introduce a SalI restriction site within the open reading frame. PCR products were joined by overlap PCR using primers porB forward/porB reverse and then introduced into pGEM-T *Easy* (Promega). Constructs were verified by sequencing. Kan resistance cassette was amplified using overlap primers porBM forward/porBM reverse with SalI recognition sites at the 5′ ends and ligated into the SalI site in *porB* in pGEM-T *Easy*. The *porB*::kan construct was amplified by PCR using primers porB forward/porB reverse. Plasmids for modifying gonococcal PorB loops were kindly provided by Hank Seifert (26). Plasmids were purified by GenElute (Sigma) according to the manufacturer’s instructions. Linearized plasmid DNA or amplified constructs were used to transform *Neisseria* (22). Transformants were identified by plating to selective media and verified by PCR and sequencing.

### Generation of anti-CFHR MAbs.

All work with animals was conducted in accordance with the United Kingdom Home Office guidelines under relevant project licenses. Expression and purification of CFH_(19,20)_, CFHR1_(4,5)_, CFHR2_(3,4)_, CFHR3_(4,5)_, CFHR4_(4,5)_, and CFHR5_(8,9)_ were described previously (7, 17). MAbs were raised against recombinant CFHR2_(3, 4)_, CFHR5_(8,9)_, and a linear peptide of CFHR4 (ENSRAKSNGM, Severn Biotech Ltd., UK) conjugated to keyhole limpet hemocyanin (Imject mcKLH, Pierce) as antigens to immunize female BALB/c mice (Charles Rivers, Margate) as previously described (17). ELISA plates (F96 maxisorp, Nunc) were coated with unconjugated CFH_(19,20)_, CFHR1_(4,5)_, CFHR2_(3,4)_, CFHR3_(4,5)_, CFHR4_(4,5)_, or CFHR5_(8,9)_ (5 μg mL^−1^, 50 μL per well) or the linear peptide of CFHR4 and screening was performed as previously described (17). MAbs used in this study are listed in Table 2.

**TABLE 2:** Antibodies

Antibody target	Antibody name	mAb/pAb	Isotype	Reference/supplier
CFH	OX-24	mAb	IgG1	29
CFHR2	HSL-2	mAb	IgG1	This study
CFHR3	HSL-1	mAb	IgG1	17
CFHR4	HSL-4	mAb	IgM	This study
CFHR5	HSL-5	mAb	IgG1	This study
PorA	Anti-meningococcal serosubtype P1.7	mAb	IgG2a	NIBSC, UK (63)
PorB	Anti-meningococcal serotype P3.15	mAb	IgG2a	NIBSC, UK (63)
PorB P.IB	H5.2	mAb	IgG2a	64
Lst	Anti-Lst	pAb		65
Mouse Ig	Goat anti-mouse immunoglobulins/HRP	pAb		Agilent, Dako
Mouse IgG	Goat anti-mouse IgG (H + L) Alexa Fluor 647	pAb		LifeTech, ThermoFisher Scientific
Mouse IgM	Goat anti-mouse IgG (H + L) Alexa Fluor 488	pAb		LifeTech, ThermoFisher Scientific

Supernatants from hybridomas grown in Gibco Protein Free Hybridoma Medium II (PFHM II) were diluted 1:1 with 50 mM sodium acetate, pH 5.0. Protein G chromatography cartridges (Pierce, ThermoFisher Scientific) were used to purify MAbs and eluted with Pierce IgG elution buffer (ThermoFisher Scientific) and quantified using a Nanodrop 2000c spectrophotometer (ThermoFisher Scientific). MAbs were isotyped using a Mouse Monoclonal Antibody Isotyping Test kit (Bio-Rad, UK).

### SDS-PAGE and Western blotting.

To examine MAb specificity, 1% normal human serum (NHS, vol/vol) was used as samples as previously described (22). PorB was detected as described previously (22), using anti-PorB MAb H5.2 (29). Protein was transferred to nitrocellulose membrane (Amersham) and detected using Goat anti-mouse HRP (Dako) (52). Coomassie brilliant blue staining was used to visualize proteins.

### Flow cytometry.

Flow cytometry for N. gonorrhoeae was performed as previously (26). For N. meningitidis the protocol had the following exceptions. Antibody incubations were for 1 h, and PBS 0.05% BSA (wt/vol) was the wash buffer. Recombinant CFHR5 (R&D) and purified CFH (Sigma-Aldrich) were also used to detect binding. Anti-CFHR MAb supernatants, purified OX-24 (10 μg mL^−1^), or purified HSL-5 (1 μg mL^−1^) were used as primary antibodies. anti-P1.7 and anti-P3.15 MAbs (NIBSC, UK) were used to quantify meningococcal PorA and PorB expression, respectively, while N. gonorrhoeae PorB was detected with MAb H5.2. Goat anti-mouse IgG-Alexa fluor 647 or goat anti-mouse IgM Alexa Fluor 488-conjugated (both Molecular Probes, Life Technologies) pAbs were used as secondary antibodies. Results were expressed as the geometric mean fluorescence (GMT, FlowJo vX software, Tree Star). Statistical significance was tested using an unpaired Student's *t* test or one-way ANOVA, as indicated (GraphPad Prism v.6.0).

### Serum survival assay.

N. gonorrhoeae was grown in GCBL with or without CMP-NANA from overnight growth on GCB agar. A total of 5 × 10^3^ CFU of bacteria diluted in Eagle’s minimum essential medium (EMEM) (Sigma) were preincubated with 4 μM CFHR5 or PBS for 15 min before incubation with 75% IgG and IgM depleted, human complement (hComplement) of (Pel-Freez LLC, USA) or heat-inactivated hComplement (HI-hComplement) for 45 min at 37°C in the presence of CO_2_. Bacterial survival was determined by plating onto GCB agar. Relative survival was calculated from samples containing no CFHR5 and incubated with HI-hComplement expressed as percentage survival. Statistical significance was tested using the two-way ANOVA multiple comparisons in GraphPad Prism v.9.0 (GraphPad Software Inc.) to compare means ± S.D. using a *P* < 0.05 cutoff for significance.
