# Supplementary material for: Pathogenic Neisseria Bind the Complement Protein CFHR5 via Outer Membrane Porins
Source: Infect Immun. 2022 Oct 4;90(10):e00377-22. doi: 10.1128/iai.00377-22 (PMC9584296; doi:10.1128/iai.00377-22)
Supplement: Supplemental file 1 — Fig. S1 to S4 and Table S1. Download iai.00377-22-s0001.pdf, PDF file, 0.3 MB [file iai.00377-22-s0001.pdf]

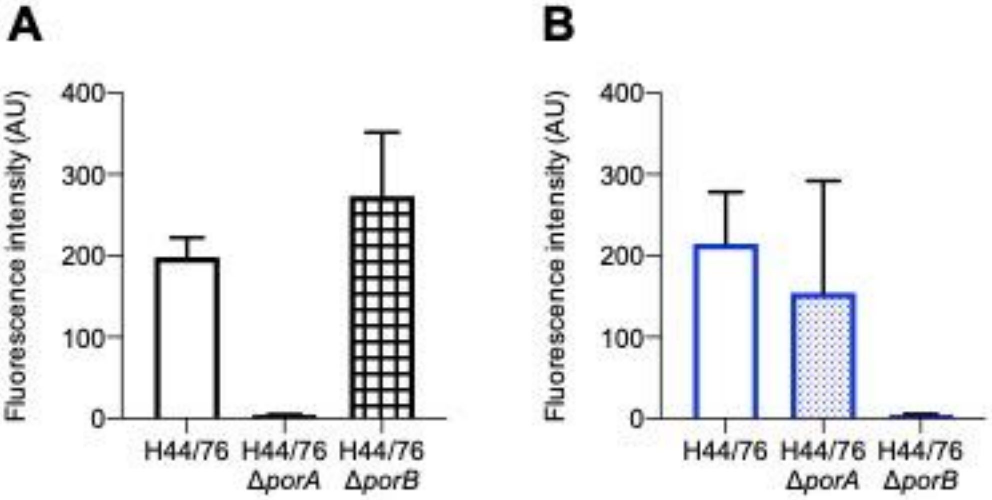

Supplementary Figure 1: Porins were successfully knocked out in H44/76 background.

Meningococcal PorA (B) and PorB (C) levels in *N. meningitidis* H44/76 strains WT, H44/76Δ*porA* and H44/76Δ*porB*, were quantified using well-characterised meningococcal serosubtype and serotype typing mAbs, respectively (NIBSC, UK; 01/514 and 02/310 respectively). There was no expression of *porA* and *porB* in H44/76Δ*porA* and H44/76Δ*porB* respectively. A non-significant increase in PorB levels was seen in H44/76Δ*porA*. Significance was analysed with one-way ANOVA. ns, $p \geq 0.05$.

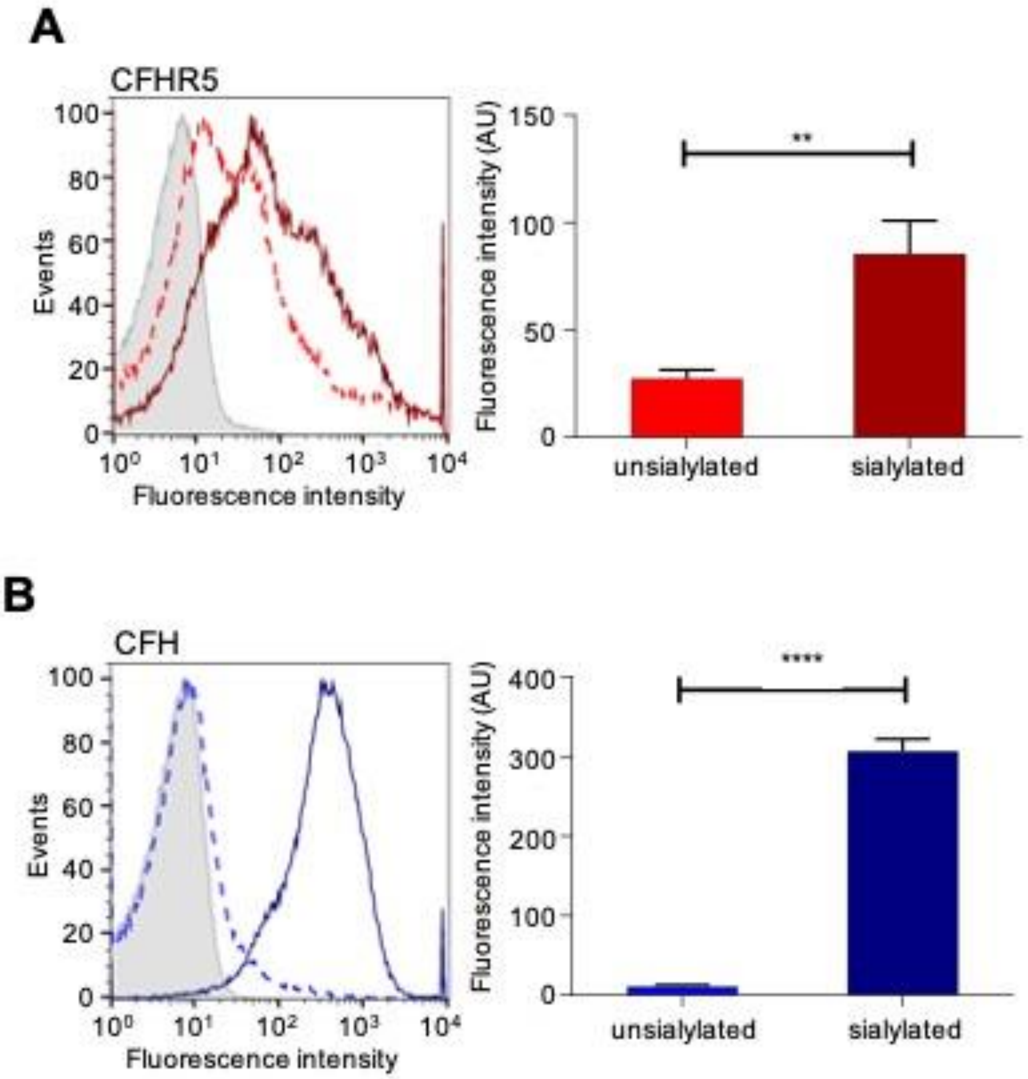

Supplementary Figure 2: Sialylation is required for CFHR5 and CFH binding to *N. gonorrhoeae*

In the presence of sialylation, *N. gonorrhoeae* FA1090 binds purified CFHR5 (A) and CFH (B) by flow cytometry. Geometric mean fluorescence intensity and s.d. (n ≥ 3 independent assays) are indicated and values were analysed with an unpaired t test. **, $p \leq 0.01$, ****, $p \leq 0.0001$.

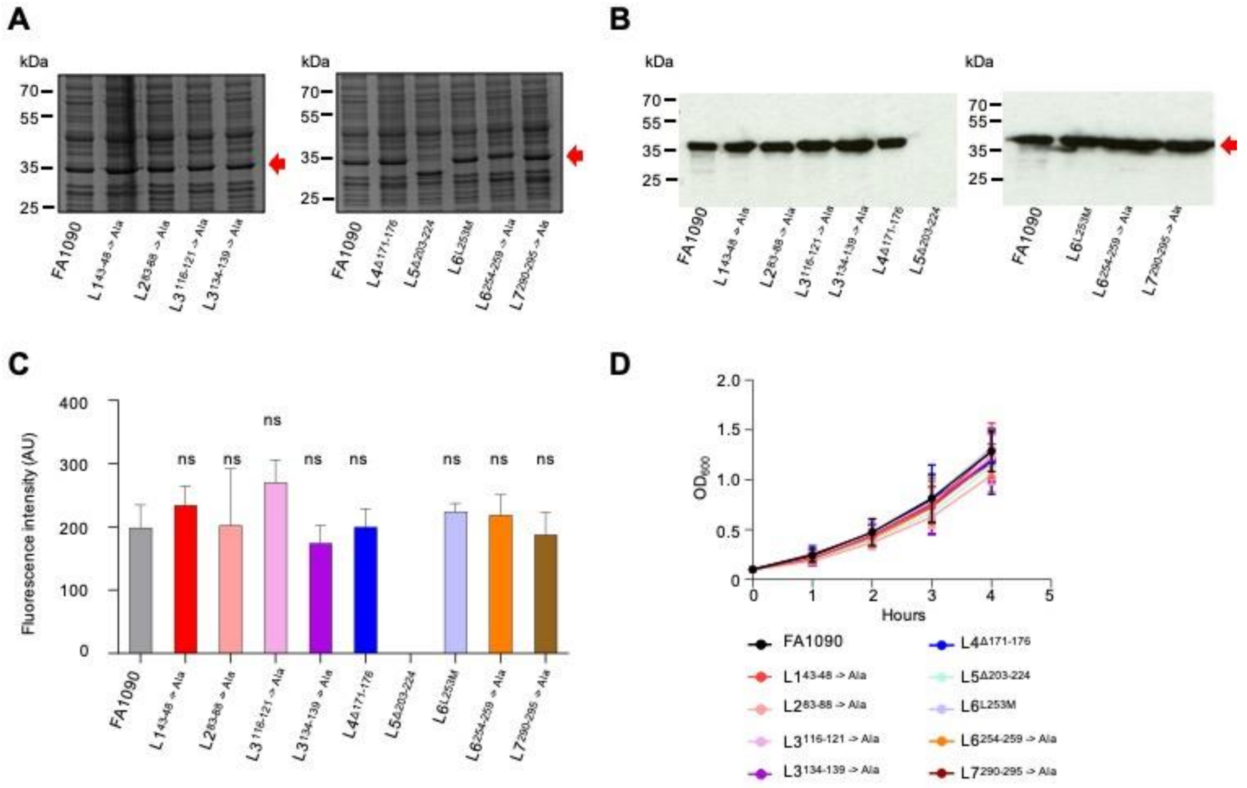

Supplementary Figure 3: Levels of *N. gonorrhoeae* P.IB and growth rate are unchanged in FA1090 P.IB loop mutants. (A) Analysis of P.IB expression levels in *N. gonorrhoeae* FA1090 by SDS-PAGE gel stained with Coomassie Blue. Molecular weight of gonococcal P.IB is approximately 37 kDa (red arrow). (B) Western blot to analyse levels of P.IB in *N. gonorrhoeae* FA1090 loop mutants, probed with a characterised anti-PorI.B mAb, H5.2. Red arrow indicates P.IB. (C) Surface P.IB levels were determined by flow cytometry. There was no significant change in P.IB levels in *N. gonorrhoeae* FA1090 and the isogenic loop mutants. No binding of the anti-P.IB antibody was detected to the L5$^{\Delta 203\text{-}224}$ loop mutant as the antibody epitope is in P.IB loop 5. Significance was analysed with one-way ANOVA, with wild-type *N. gonorrhoeae* FA1090 as the control; ns, $p > 0.05$. Geometric mean fluorescence intensity was used to determine antibody binding. (D) Viability of FA1090 and isogenic loop mutants during growth in liquid GCB media. OD_{600} values

are shown as mean ± s.d. ($n \geq 3$), and significance was analysed with one-way ANOVA, with wild-type *N. gonorrhoeae* FA1090 as the control; ns, $p \geq 0.05$.

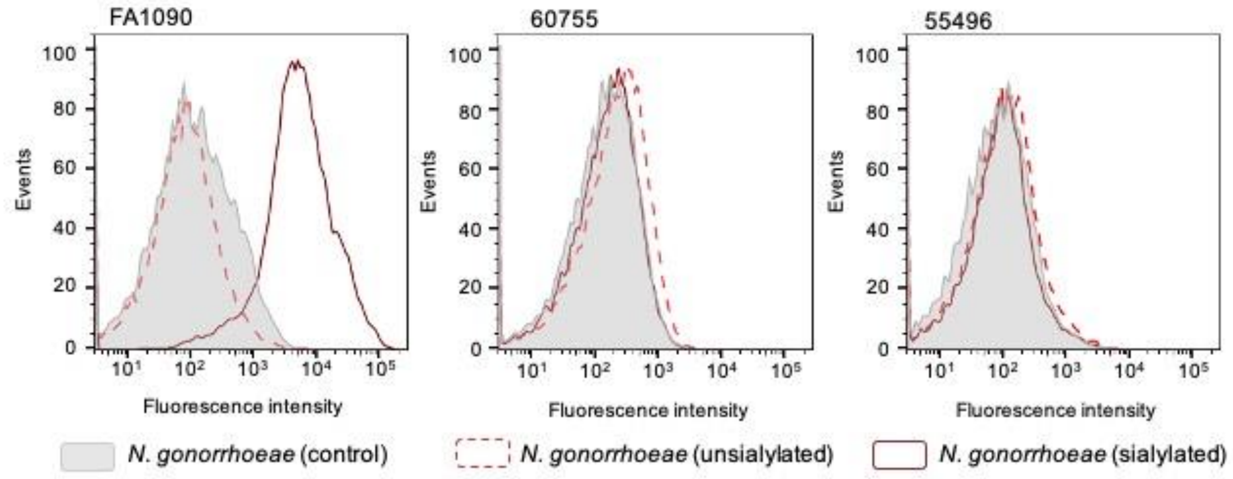

Supplementary Figure 4: Clinical isolates expressing P.IA do not bind CFHR5. CFHR5 binding to clinical isolates 60755 and 55496 expressing P.IA were quantified by flow cytometry. FA1090 was included as a positive control. No binding (indicated by shift in fluorescence) was observed in the presence or absence of sialylation.

PRIMER NAME	SEQUENCE (5' TO 3')	AMPLIFICATION PRODUCT
**LS1_F**	ACGGTATCGATAAGCTTGATATCGAATTCAAACC GCACCGCCGCGTTCAAATCTAT	500bp upstream of *lst*
**LS1_R**	ATCGTCATCGGGGGTATCGGATCCAAGGGCAAA ATGGCGGAGTAAGTAAGGCAAAAATCA	500bp upstream of *lst*
**LS2_F**	CTTATCTCTTTTCAATAGCTATAAATTATTTAATAA GTAAGTTAAGGGATGCATAAACATCCCTAAAAAC TCCATTCCGAC	500bp downstream of *lst*
**LS2_R**	GCTTACATAAACAGTAATACAAGGGGTGTTATGT CGGCAGTTTTGACATC	500bp downstream of *lst*
**ERY_F**	GCCCTTGGATCCGATACCCC	Erythromycin cassette (E. coli RE642)
**ERY_R**	GTTTATGCATCCCTTAACTTAC	Erythromycin cassette (E. coli RE642)
**PB1_F**	CTACAGAGTTCTTGAAGTGGTGG	A fragment of the pBluescript
**PB1_R**	TGAATTCGATATCAAGCTTATCGA	A fragment of the pBluescript
**PB2_F**	AACACCCCTTGTATTACTGTTTATGTAAGC	A fragment of the pBluescript
**PB2_R**	AGTTAGGCCACCACTTCAAGAAC	A fragment of the pBluescript
**PORB FORWARD**	CCCGTTCCGAAAGAAACCACGTG	500bp upstream of *porB*
**PORB REVERSE**	CCCGATAAATGCCGCAACCTCA	500bp downstream of *porB*
**PORBM FORWARD**	**GTCGAC** AGGCCGGCAA ATTCGGGAGA TA	*porB*/ kan cassette overlap primer (Sal*I*)
**PORBM REVERSE**	**GTCGAC** CGCTTAACGA CAATGCAGGC AG	*porB*/ kan cassette overlap primer (Sal*I*)

Supplementary Table 1: List of primers used in this experiment
